# Occurrence of Metastases in Differentiated Thyroid Carcinoma Patients: A Retrospective Study in Morrocco Covering 10 Years of Follow-Up

**DOI:** 10.7759/cureus.78176

**Published:** 2025-01-29

**Authors:** Hajar Tabiti, Abdel Amide Gbadamassi, Karima Bendahhou, Zakaria Oussafrar, Amal Guensi

**Affiliations:** 1 Laboratory of Cellular and Molecular Inflammatory, Degenerative and Oncologic Pathophysiology, Université Hassan II de Casablanca, Casablanca, MAR; 2 Department of Nuclear Medicine, Centre Hospitalo-Universitaire Ibn Rochd, Casablanca, MAR; 3 Department of Epidemiology and Public Health, Centre Hospitalo-Universitaire Ibn Rochd, Casablanca, MAR

**Keywords:** differentiated thyroid carcinoma, fdtc, mestatasis occurrence, predective factors, thyroid cancer

## Abstract

Introduction: Distant metastases in differentiated thyroid carcinoma (DTC) are uncommon but significantly impact patient outcomes. The disease-specific mortality rates are notably higher among patients with metastases, reflecting the aggressive nature of this condition. This study aimed to identify predictive factors for the occurrence of distant metastases in Moroccan patients treated for DTC.

Material and methods: This was a retrospective cohort study that included patients diagnosed with DTC between 2004 and 2012 and followed up at the nuclear medicine department of Ibn Rochd Hospital, Casablanca, Morocco. All patients diagnosed with DTC who did not have metastasis at the diagnosis were included with a mean follow-up time of 10.4 years. The Kaplan-Meier method was used to study the occurrence of metastases at 10 years. The diagnosis of metastases was confirmed by iodine scintigraphy and fludeoxyglucose F18 (18F-FDG) PET/CT.

Results: A total of 1,357 patients were included in the study. Women represented 89.5% of the study population. The mean age was 44, with a range of 14-85 years. The most frequent histological type was papillary thyroid cancer, accounting for 93.5% of cases. During follow-up, 6.2% of patients developed metastases. The mean time from diagnosis to metastasis was 4.27 years. Lung (46.0%) and bone (38.0%) were the most common sites of distant metastases.

The study of factors predictive of the occurrence of metastases showed that age ≥ 55 years, male gender, follicular histological type, extra-thyroidal extension, vascular invasion, tumor size > 4 cm, detectable thyroglobulin level, disease stages (≥II), high risk of relapse, and the presence of cardiovascular comorbidities were associated with the occurrence of metastases.

Conclusion: The identification of factors predictive of the occurrence of metastases offers a valuable opportunity to stratify patients according to their level of risk. This stratification makes it possible to optimize management through an adapted and more aggressive therapeutic approach for high-risk patients while ensuring close monitoring, particularly during the first five years following diagnosis.

## Introduction

Thyroid cancer is the most common endocrine malignancy worldwide [1]. Histologically, differentiated thyroid carcinoma (DTC), which develops from the epithelial cells of the thyroid gland, is the most common [2]. It is represented by papillary carcinomas (PTC), follicular carcinomas (FTC), and oncocytic carcinomas [3].

According to the WHO, thyroid cancer is the ninth most common cancer in the world [4], with an incidence that has increased in recent decades throughout the world [5]. In Casablanca, Morocco, the incidence standardized to the world population is eight per 100,000 inhabitants, making it the second most common cancer in women after breast cancer [6].

Distant metastases are a rare but pejorative event in DTC, with a prevalence of around 5% [7]. It should be noted that the majority of thyroid carcinoma metastases are asymptomatic and discovered only during systemic surveillance or a complete metastatic work-up of a malignant thyroid nodule [8]. Although the majority of DTCs behave indolently compared with most other cancers, 1-9% of DTC patients present with locally advanced cancer with distant metastases at the time of diagnosis. During follow-up, 7-23% develop distant metastases after postoperative treatment [7]. Distant metastases significantly impact survival outcomes in patients with DTC, particularly in cases of follicular thyroid carcinoma and poorly differentiated or advanced-stage papillary thyroid carcinoma. The lungs and bones are the most common sites of distant metastases, and their presence is associated with a drastic reduction in survival rates. The 10-year disease-specific mortality rate for patients with distant metastases has been estimated at 75%. However, less than 5% of patients without metastases die from the disease [9,10].

Individual prognosis is influenced by several factors, including the distribution and number of metastatic sites involved, serum thyroglobulin (Tg) level, histological type, and age [11]. While several studies have reported prognostic factors for survival, local recurrence, and mortality in DTC, there remains a lack of information regarding factors predictive of metastasis in patients with DTC.

The evidence available to clinicians to assess the risk of metastasis in patients diagnosed with DTC is relatively limited. Therefore, it is important to identify patients at risk of metastasis early in the treatment process in order to provide meaningful evidence for optimizing their survival. The purpose of this study is to identify predictive factors for the occurrence of distant metastases in patients followed up for DTC in Morocco, with the aim of ensuring optimal management.

## Materials and methods

This was a retrospective cohort study that included all patients diagnosed with follicular DTC between 2004 and 2012, having been followed up for a minimum of 10 years at the Nuclear Medicine Department of Ibn Rochd Hospital, Casablanca, Morocco. Exclusion criteria were the presence of metastases at the time of diagnosis and lack of detailed information in the patient's medical record. The study was approved by the Ethics Committee of CHU Ibn Rochd (approval n° 14/22). This was based on: (i) Law 28-13 of 17/09/2015 concerning the protection of individuals participating in biomedical research, and (ii) the decision of the Minister of Health No. 02/DRC/00 of 03/12/2012, related to biomedical research.

Data collection and assessment

Data collected from medical records included patients' sociodemographic data (age, gender), tumor characteristics (histological type, size, vascular invasion, extra-thyroid extension, lymph node invasion, stage of disease according to the American Joint Committee on Cancer (8th ed.) classification [12], level of risk of recurrence according to the recommendations of the American Thyroid Association (ATA) 2015 [13], treatment received (surgery, radioiodine (RAI) therapy, thyroid hormone replacement), follow-up (thyroid-stimulating hormone (TSH), Tg, anti-thyroglobulin antibodies, cervical ultrasound, scintigraphy, and 18 FDG PET/CT), response to treatment (excellent response, biochemical incomplete disease, persistent disease), and duration between diagnosis and occurrence of metastases.

Responses to treatment were classified according to the ATA 2015 guidelines as follows: patients with an excellent response had NED, with suppressed Tg < 0.2 ng/mL or TSH-stimulated Tg < 1 ng/mL, no detectable antibodies, and no structural disease on imaging. Biochemical incomplete response was defined by suppressed Tg ≥ 1 ng/mL or TSH-stimulated Tg ≥ 10 ng/mL, or rising anti-Tg antibody levels without structural disease on imaging. A structural incomplete response indicated structural evidence of disease on imaging. Indeterminate response included suppressed Tg < 1 ng/mL or TSH-stimulated Tg < 10 ng/mL with declining or stable anti-Tg antibody levels. Persistent disease was defined as either a biochemical or structural incomplete response.

The diagnosis of metastases was confirmed by iodine scintigraphy and fludeoxyglucose F18 (18F-FDG) PET/CT.

Data analysis

Data were processed and analyzed using jamovi software version 2.3.17 (The jamovi Project, https://www.jamovi.org). Quantitative variables were summarized as median, mean, and standard deviation (SD), while qualitative data were described using frequencies. The Kaplan-Meier method was employed to estimate the probability of metastasis at 10 years, with the starting point being the date of histological confirmation and the endpoint being the date of metastasis occurrence. Survival curves' divergence was assessed using the log-rank test to determine statistical significance between independent variables and metastasis occurrence. Detailed statistical outputs were reviewed to ensure robustness and accuracy in the findings. The value of p considered statistically significant in our study is p<0.05.

## Results

A total of 1357 patients were included in the study, with regular follow-up over 10 years. The mean age of patients was 44 ± 6.3 years, with the age group of ≥55 years being the least represented (21.4%). The majority of the cohort was of the female sex (89.5%), with a female/male sex ratio of 8.6. The comorbidities most frequently encountered in the patients were diabetes mellitus (6.7%) and arterial hypertension (5.9%). Histologically, tumor size was greater than 4 cm in 11.5% of patients, with follicular carcinoma being the least frequent histological type (6.5%). The tumor was multifocal in 29% of cases, encapsulated in 21.5%, with extra-thyroid extension and vascular invasion in 5.4% and 6.1%, respectively. A total of 30 patients had lymph node involvement. The distribution of patients according to disease stage revealed that 3.8% were at stage II, 0.6% at stage III, and 0.5% at stage IV. According to the 2015 ATA classification, 8.8% of patients were at high risk of disease relapse (Table 1).

**Table 1:** Patient characteristics PTC: papillary carcinoma; FTC: follicular carcinoma; Tg: thyroglobulin; RAI: radioiodine therapy

Characteristics	Frequency	Percentage
Age (mean±SD = 44 ± 6.13; range: 14-85 years)		
<55 years	1067	78.6
≥55 years	290	21.4
Sex		
Male	142	10.5
Female	1215	89.5
Diabetes		
Yes	92	6.7
No	1265	93.2
High blood pressure		
Yes	80	5.9
No	1277	94.1
Histological type		
PTC	1268	93.5
FTC	89	6.5
Tumor size		
≤4 cm	921	68.5
>4 cm	144	11.5
Unknown	272	20.0
Encapsulated		
Yes	292	21.5
No	1065	78.5
Multifocal		
Yes	394	29.0
No	963	71.0
Extra-thyroidal extension		
Yes	73	5.4
No	1285	94.6
Vascular invasion		
Yes	83	6.1
No	1274	93.9
Postoperative serum Tg		
Undetectable	519	38.0
Detectable (>1 ng/m and≤10 ng/ml)	456	34.0
>10 ng/ml	382	28.0
Cervical nodes at diagnosis		
Yes	30	2.2
No	1327	97.8
Stage		
I	1292	95.1
II	52	3.8
III	8	0.6
IV	6	0.5
Risk groups		
Low Risk	1149	82.7
Intermediate risk	112	8.5
High risk	96	8.8
RAI ablation		
Yes	1161	85.5
Average delay: 18 months; Average dose: 3.959 GBq		
Number of treatments		
1	1107	95.0
2	44	4.0
> 2	10	1.0
Tg level after radioiodine		
Undetectable	964	83.0
Detectable (>1 ng/m and≤10 ng/ml)	144	12.5
>10 ng/ml	53	4.5

All patients underwent total thyroidectomy. Serum Tg levels, measured after one month's thyroid hormone withdrawal, were detectable in 62% of patients. It was greater than 10 ng/ml in 28% of patients. All patients received hormone replacement therapy and 85.5% received RAI ablation. Among these patients, 5% received more than one course of RAI ablation, and the mean dose was 3.95 GBq (min 3.7 GBq; max 27.75 GBq). The mean time between total thyroidectomy and the first dose of RAI ablation was 16 months, with a range of one month to eight years. After treatment, Tg levels were detectable (>10ng/ml) in 4.5% of patients (Table 1).

The mean follow-up time was 10.4 years. During follow-up period, it was seen that 85 (6.2%) patients developed distant metastases. The mean time from diagnosis to metastasis was 4.27 years ± 2.9 years (minimum 26 days, maximum 10 years). Lung and bone were the most common sites of distant metastases, being 46% and 38%, respectively (Table 2).

**Table 2:** Metastasis localization (N=85) Of the 1357 patients included in the study, 85 (6.25%) showed metastases.

Location	Frequency	Percentage
Lungs	39	46.0
Bone	32	38.0
Brain	9	10.6
Bone and lungs	5	5.8

**Figure 1:**
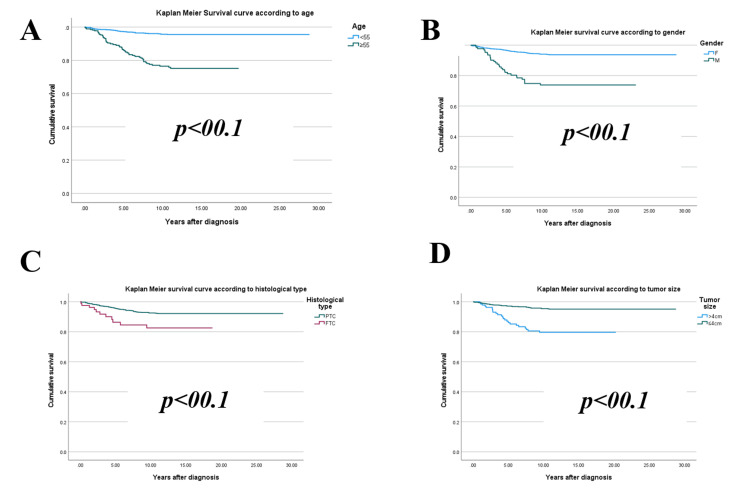
Kaplan-Meier curve of metastasis occurrence during follow-up according to (A) age, (B) gender, (C) histological type, (D) tumor size.

**Figure 2:**
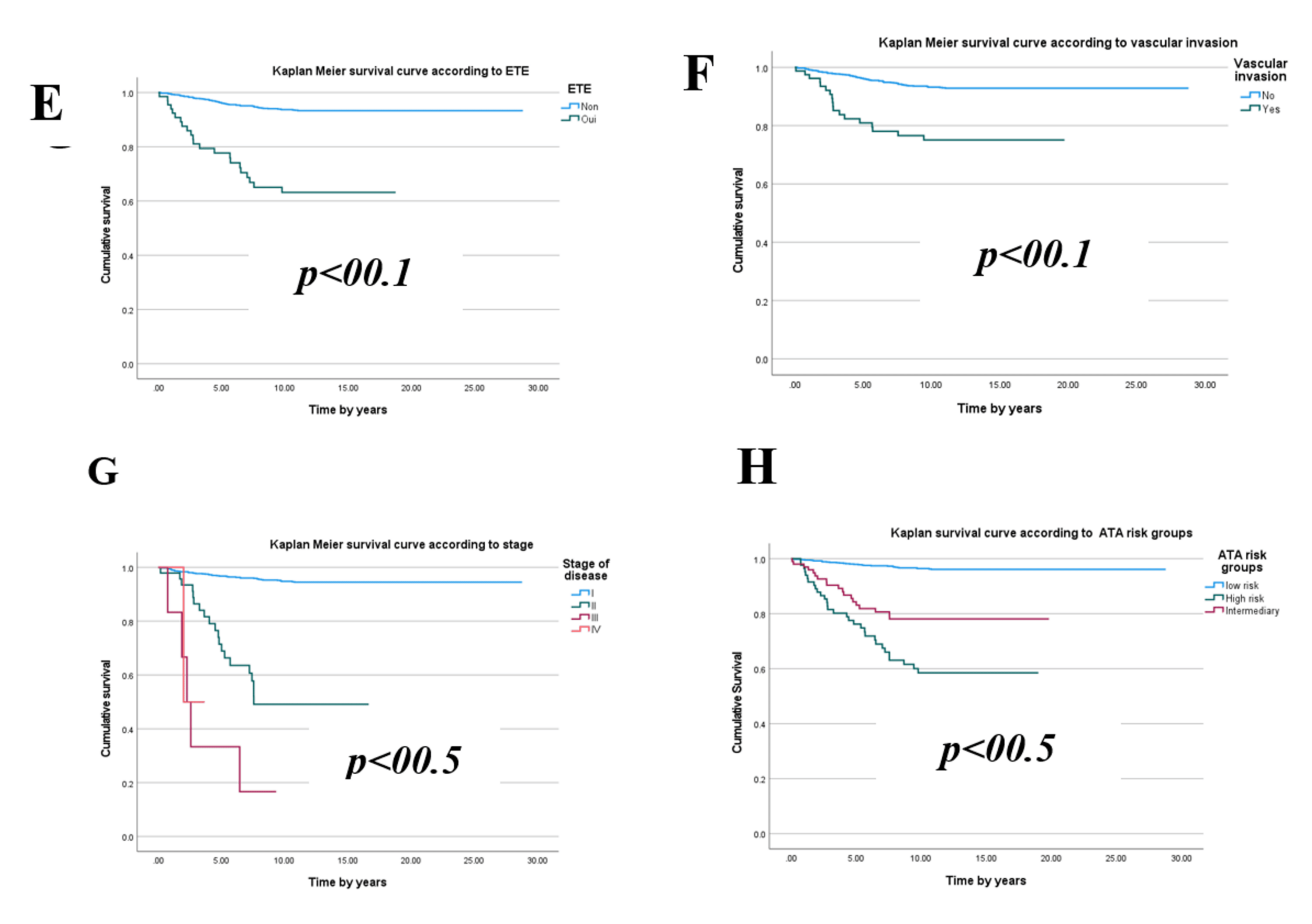
Kaplan-Meier curve of metastasis occurrence during follow-up according to (E) extra-thyroidal extension (ETE), (F) vascular invasion, (G) disease stage, (H) American Thyroid Association (ATA) risk groups.

**Figure 3:**
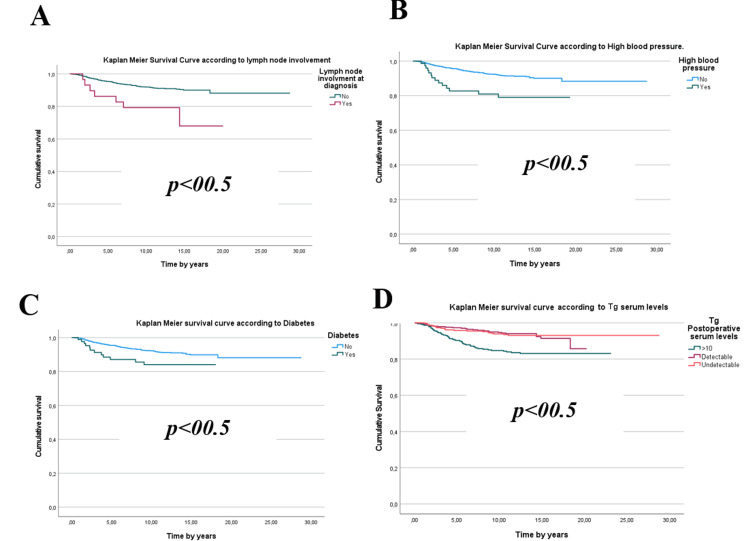
Kaplan-Meier survival curves according to (A) lymph node involvement, (B) high blood pressure,(C) diabetes, and (D) thyroglobulin (Tg) postoperative serum levels

An analysis of sociodemographic, clinical, and histopathological characteristics highlights several factors significantly associated with the development of metastases in patients. Key contributors include age≥ 55 years, male gender, tumor size exceeding 4 cm, follicular histological subtype, extra-thyroidal extension, vascular invasion, lymph node involvement at diagnosis, postoperative serum Tg levels ≥ 10 ng/ml, advanced disease stages (≥ II), high disease risk, and cardiovascular comorbidities (p < 0.05) (Figures 1-3).

Kaplan-Meier survival curve analyses reveal that male patients aged over 55 with follicular**,** multifocal thyroid tumors larger than 4 cm, exhibiting extra-thyroidal extension and vascular invasion, face a significantly elevated risk of metastasis. Similarly, individuals with postoperative or post-radioactive iodine ablation Tg levels exceeding 10 ng/ml, as well as those with advanced disease stages (II, III, or IV) or a high relapse risk, also demonstrate a marked vulnerability.

These differences manifest over distinct timeframes depending on the influencing factors. On average, metastases emerge after 4.27 ± 2.9 years, but high-risk groups often exhibit divergence as early as the fourth year. Some unfavorable indicators, such as advanced age, male gender, or elevated Tg levels, contribute to earlier deviations (within one to two years). In contrast, other factors like tumor size and advanced disease stages yield more pronounced effects around three to five years.

## Discussion

Ethnic variation in thyroid cancer is well-documented, with different groups and regions showing deviations in trends and prognostic factors [14]. For Arab and African populations, the current study provides essential data on the long-term outcomes of DTC and its determinants. National studies are crucial for uncovering region-specific genetic, environmental, and healthcare influences. The findings of the current study offer valuable insights into Moroccan patients with DTC, providing a foundation for future national and regional research.

Thyroid cancer, the most common endocrine tumor, typically has a favorable prognosis, with 90% of cases showing good outcomes. However, distant metastases remain a significant negative prognostic factor, contributing to an estimated overall 10-year mortality rate of 75% [9]. In contrast, patients without metastases experience a mortality rate of less than 5% [10]. This underscores the importance of early detection and monitoring, particularly in populations with varying genetic and environmental factors, as highlighted by our study.

The present study revealed that 6.2% of Moroccan patients with DTC developed metastases during a 10-year follow-up. Age ≥ 55 years, male gender, follicular histological type, extra-thyroidal extension, vascular invasion, tumor size > 4 cm, Tg level > 10 ng/ml, disease stage ≥ II, high-risk disease, and the presence of cardiovascular comorbidities were identified as significant risk factors for the occurrence of metastases.

Globally, DTC predominantly affects women. Female patients are more likely to present with less aggressive histological subtypes, which contributes to higher rates of complete remission compared to males. Male sex is frequently associated with a poorer prognosis, as men are often diagnosed at later stages with more aggressive forms of DTC [15]. This study corroborated these findings, identifying male sex as a significant predictor of metastasis occurrence (p<0.001).

Age and disease stage are key prognostic factors in DTC. In our study, patients aged ≥ 55 years, as well as those in advanced stages, were more likely to develop metastasis, which aligns with findings from Saudi Arabia [14]. In that study, age ≥ 55 years was also identified as a major negative prognostic factor for DTC, influencing survival. The stage at diagnosis further compounded these risks, with advanced-stage patients demonstrating significantly worse survival outcomes. These findings reinforce the critical role of early diagnosis and timely treatment in improving patient prognosis and highlight the need for targeted strategies for older patients and those diagnosed at more advanced stages.

All histological types of DTC can give rise to metastases, and our study revealed that FTC was associated with a greater risk of distant metastases compared to PTC. Lung and bone metastases represented the most frequent sites of distant metastasis. Our results are similar to those reported in the literature [15-17]. The lungs are the most frequent site of distant metastases, responsible for reduced survival, followed by bone (13-21% at 10 years) [18,19]. The probable explanation is that FTC spreads more easily via the bloodstream to distant organs due to its major tendency to invade blood vessels [20].

Serum Tg is the specific DTC biomarker pivotal in the follow-up of these patients. Progressive elevation of serum Tg in DTC after total thyroidectomy, whether followed by RAI ablation or not, is highly predictive of recurrence or metastasis [21]. In studies focusing on Tg levels, it has been reported that Tg levels are not only a poor clinical prognostic factor but also predictive of recurrence and metastasis [22]. In the current study, all patients who developed metastases during follow-up had a detectable Tg level (>10 ng/ml).

Extensive vascular invasion (more than four vessels), massive capsular invasion, tumor size > 4 cm, and multifocal tumors are poor prognostic factors that increase the risk of recurrence and distant metastasis in the vast majority of studies [23,24], corroborating the results of the present study.

Although this study examined a large cohort of patients with DTC, certain limitations must be acknowledged. Firstly, the data used in this study were obtained retrospectively, and the study was conducted in a single center. These factors limit the generalizability of the results to other populations or regions, as variations in demographics, healthcare systems, and genetic profiles may exist. Secondly, we did not analyze histological subtypes of thyroid carcinoma and were unable to identify patients with tumors of more aggressive behavior (e.g., tall-cell variant and insular variant), which could have had an impact on the plurality of metastases. Moreover, this study focused mainly on demographic, histological, and clinical risk factors associated with the occurrence of metastases in patients followed for DTC without explicitly exploring the influence of genetic factors, such as mutations, which may coexist with the predictive factors identified.

The study by Justiniano et al. confirmed the presence of mutations in known tumor factors (*BRAF* and *RAS*) in metastatic samples [25]. In addition, they found a co-occurrence of mutations in functional variants of the *ATM* and *ERCC4* genes responsible for DNA damage repair in metastatic lesions. Future research should consider integrating genetic analyses to elucidate the interaction between genetic predispositions and identified predictive factors to better understand the occurrence of metastasis during follow-up in DTC patients.

## Conclusions

This study provides valuable information on the characteristics of follicular DTC patients at risk of developing metastases during follow-up. Findings on the factors that lead to metastasis during follow-up are essential for clinicians to identify high-risk patients and tailor follow-up and treatment plans accordingly. Such predilection at diagnosis is of great clinical value, as it can prevent metastasis. These results therefore have the potential to improve patient care and management, while identifying further avenues for research and development of new treatment options.
